# Learning About Gene Regulatory Networks From Gene Deletion Experiments

**DOI:** 10.1002/cfg.220

**Published:** 2002-12

**Authors:** Thomas Schlitt, Alvis Brazma

**Affiliations:** European Bioinformatics Institute Wellcome Trust Genome Campus Hinxton, Cambridge CB10 1SD UK

## Abstract

Gene regulatory networks are a major focus of interest in molecular biology. A crucial question is how complex regulatory systems are encoded and controlled by the genome. Three recent publications have raised the question of what can be learned about gene regulatory networks from microarray experiments on gene deletion mutants. Using this indirect approach, topological features such as connectivity and modularity have been studied.


**Comparative and Functional Genomics**
*Comp Funct Genom* 2002; **3**: 499–503.
Published online in Wiley InterScience (www.interscience.wiley.com). **DOI:** 10.1002/cfg.220

**Conference Review**

### Learning about gene regulatory networks from gene deletion experiments

Thomas Schlitt* and Alvis Brazma
*European Bioinformatics Institute, Wellcome Trust Genome Campus, Hinxton, Cambridge CB10 1SD, UK*

**Correspondence to: Thomas Schlitt, European Bioinformatics Institute, Wellcome Trust Genome Campus, Hinxton, Cambridge CB10 1SD, UK. E-mail: schlitt@ebi.ac.uk*

Received: 9 September 2002
Accepted: 14 October 2002

### Abstract

**Gene regulatory networks are a major focus of interest in molecular biology. A crucial question is how complex regulatory systems are encoded and controlled by the genome. Three recent publications have raised the question of what can be learned about gene regulatory networks from microarray experiments on gene deletion mutants. Using this indirect approach, topological features such as connectivity and modularity have been studied. Copyright © 2002 John Wiley & Sons, Ltd.**

**Keywords:** **gene regulatory networks; power-law; yeast**

The collection of large-scale data such as genome sequences, transcriptome, proteome and interactome data stimulates the development of methods to predict the functions of genes and proteins. The genome projects helped to compile 'parts-lists' of the cell; one remaining big task is to find modelling approaches to identify key regulatory molecules and unravel regulatory networks directly from experimental data. For small molecular systems such as λ-phage, mathematical and computational models have been developed: techniques such as Boolean networks, differential equations or mixed models allow simulations and predictions leading to experimental verification [16]. These techniques have been used successfully for well-studied model systems (e.g. in [10]). Nevertheless, the scaling of these techniques to the size of eukaryotic genomes is difficult — not the least because of the limited information available for many less well known genes despite of the increasing number of genome-scale, high-throughput experiments that have been published. Therefore, one has to start with simpler questions, aiming to describe only the 'wiring' of the genes and regulators.

Three recently published articles by Featherstone and Broadie [4], Wagner [19] and Rung *et al.* [13] address the question of what can be learned about gene regulatory networks from microarray data. All three articles are based on the same comprehensive microarray dataset, studying the effects of over 270 gene deletions in yeast by Hughes *et al.* [6]. The set of deletion mutants were selected to represent a wide range of cellular roles in yeast. One problem with microarray technology is how to be sure whether or not the measured change in gene expression is significant. Hughes *et al.* provide an error-model that takes wild-type vs. wild-type comparisons into account. This allows them to distinguish genes that have a high natural variability in their expression levels from those genes that are much more tightly controlled. They normalize the expression data for all genes, so only one threshold is to be chosen, which applies to all measurements for all genes. Depending on this threshold, changes in expression are required to be larger or smaller, to be considered significant. This error-model allows the discretization of the expression levels to 'significantly upregulated', 'significantly downregulated' and 'no significant change'.

All three studies make use of this error model to give an alternative representation of the data in form of a graph [4,13,19]. Graphs are a well-established way to represent information in computer science and mathematics [3]. Graphs consist of *nodes*, also called *vertices*, often represented by boxes or circles and *edges*, which are connections

Copyright © 2002 John Wiley & Sons, Ltd.

between the nodes, represented by lines connecting the boxes (see Figure 1). The nodes are used to represent entities like genes; the edges are used to represent relationships between these entities. The edges can be undirected or directed, depending on the type of relationship they are representing. Directed edges are called *arcs*. Thus, edges can be used to represent 'two-way' relationships, e.g. 'protein A and protein B bind to each other', whereas arcs can be used to represent 'one-way' relationships, e.g. 'protein A activates protein B'. *Undirected graphs* are graphs consisting of nodes and edges whereas *directed graphs* are graphs consisting of nodes and arcs. Graphs have been used to represent all kinds of networks. Recent biological examples are protein–protein interaction networks, where nodes represent proteins, and edges represent the physical interaction between proteins [9,15]. A *path* is an ordered list of edges (or arcs) that connect two nodes. The *path length* in this context is the number of edges (arcs) you have to 'walk along' to get from one node to the other. If there is an edge between two nodes, i.e. the path length between the two nodes is 1, we will call this a *direct* connection. If the path length between two nodes is larger than 1 we will call it an *indirect* connection. A *component* (or *subnet*) of an undirected graph is a subgraph, where all nodes are connected by paths; in a directed graph we can use the same definition if we ignore the directionality of the arcs. The *diameter* of a graph is the average length of the shortest paths between any two nodes in the graph. The *degree* of a node is the number of adjacent edges. In a directed graph it is useful to make a distinction between the *indegree*, the number of arcs pointing to a given node, and the *outdegree*, the number of arcs pointing from a given node to other nodes. Nodes with very high degrees are sometimes called *hubs*. The representation in the form of graphs allows the use of powerful algorithms to examine large datasets efficiently, e.g. to find the shortest path between two nodes.

Interestingly, as we will discuss in the following paragraph, common topological properties have been identified among many large networks as different as the Internet and protein-interaction networks. In a typical random network the distribution of edges resembles the Poisson distribution. The majority of the nodes have roughly the same degree, of about the average degree over all nodes. But in many real networks the degree of nodes varies considerably, with some nodes having a very high degree, but most nodes having a small degree. The degree distribution has a power-law tail: the probability $p(k)$ of a random node to have a particular degree $k$ follows: $p(k) \sim k^{-\gamma}$ [1]. One example of networks with this degree distribution is the World Wide Web, with nodes representing home pages, and edges representing links between home pages. Similarly, this degree distribution was also found for protein-interaction networks [12]. Another characteristic property of these networks is the *small-world* behaviour. This property is not exclusively found in graphs with power-law-distribution. It refers to the fact that the average distance between two nodes is usually small. One example for a small-world network is the social network of acquaintances between people in the USA, which has a typical path length of six between any two persons [1].

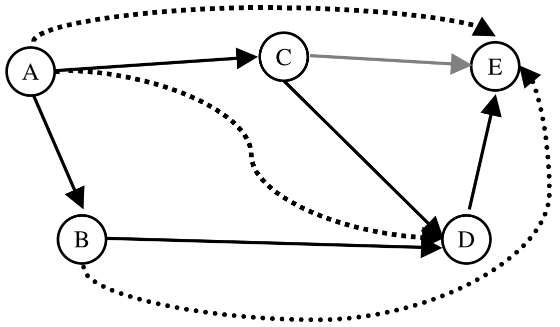

**Figure 1.** Graphical representation of a graph. Solid arcs like that from node A to node B indicate direct connections. The arc between C and E can be circumvented by an indirect path from C to E via D. The dashed arcs indicate that there are indirect connections for which there are no direct connections in the graph

The graph models used by all three groups [4,13,19] represent genes as nodes. The deleted genes are connected by arcs to all other genes showing significant differences in expression in the particular deletion mutants.

We will focus first on the studies by Featherstone and Broadie, and Rung *et al.*, because they used similar methods for the graph construction: both groups use the data published by Hughes *et al.* directly to construct graph models and study their properties. Whereas the first group built an undirected graph for one particular threshold of the expression values, Rung *et al.* examined directed graphs resulting from a wide range of

Copyright © 2002 John Wiley & Sons, Ltd. 

thresholds. Both groups use edges (arcs) to connect the mutated gene with all genes that show a significantly different expression in the particular deletion mutant in comparison to the wild-type strain.

Featherstone and Broadie, and Rung *et al.*, find a degree distribution which follows a power-law, with $\gamma$ between 0.7 (Featherstone and Broadie) and $\sim$1 (Rung *et al.*). This is considerably smaller than the range of 1.5–3 observed for other networks (see [7] and, for more examples from physics, sociology and biology [1]), meaning there are relatively more nodes with high degrees. About 50% of all edges are connected to only 5–10% of all nodes. At the significance level studied by Featherstone and Broadie, 18 genes account for about 50% of the edges. Most of these 18 genes belong to the functional groups *protein synthesis* and *regulation of transcription*. Similar results were found by Rung *et al.*, who additionally examined the median outdegree and median indegree of functional groups. The functional groups with the highest median outdegrees include regulatory functions such as *RNA turnover*, *cell stress* and *meiosis*, whereas the groups with the highest median indegrees are involved in *metabolism* (of amino acids and nucleotides). This property was found to be stable over a wide range of significance cut-offs.

One of the main problems in using the dataset from Hughes *et al.* is to make the distinction between direct effects of the gene deletions and indirect effects. Many proteins are involved in several cellular functions, and compensatory regulation might occur to correct the effects of the mutation. If a particular enzyme is deleted, a metabolite might be missing in the cell. As a result, other enzymes utilizing this metabolite might be transcribed less, although there is no direct influence of the first enzyme on the transcriptional control of the second. In graph representation, this means there is both an indirect and a direct connection between two nodes where only an indirect connection should be (see Figure 1). With the complex interplay of regulatory processes at the level of genes, mRNAs, proteins and metabolites, it is not clear how to construct a 'pure' gene regulatory network. Furthermore, only one growth condition was tested and, if the deleted gene is not crucial for the particular condition, little effect is likely to be found.

A major focus of the study by Wagner is to deal with differences between direct and indirect effects. Therefore Wagner chose a different method for graph construction: graphs constructed from the Hughes data using a similar approach to Featherstone and Broadie, and Rung *et al.* (using significance cut-offs ranging from 2 to 5) are used to estimate the average degree of the nodes. Wagner then generates two kinds of random networks, using a degree distribution resembling either the Poisson distribution or power-law. For the networks with power-law distribution, he chose a probability distribution proportional to $k^{-\gamma}$, with $\gamma = 2$. Generating his own networks gives him the advantage of knowing all direct paths and he can thus selectively add indirect paths. Therefore, he can calculate the average degree of the nodes in the randomly generated networks, excluding or including indirect connections. For further analysis he subsequently chooses the randomly generated networks that have an average degree of the nodes similar — when including indirect connections — to the graphs constructed from Hughes data. These networks, without the indirect connections, are then the focus of his study.

Wagner's results suggest the expectation of about 140–350 components (subgraphs) containing more than one gene each. Considering the large error margins and statistical assumptions, he argues in favour of a modular organization of the network and against 'global connectedness of genes and pervasive pleiotropy' [19]. This might be in accordance with Thieffry *et al.*, who studied a literature-derived regulatory network of *Escherichia coli*. They report a 'rather loosely interconnected structure' [17].

On the one hand, modular organization for gene regulatory networks has been proposed, defining a module as 'a discrete entity whose function is separable from those of other modules' [5]; but different interpretations of the term 'module' exist [18]. Modules are thought to be evolutionarily advantageous if they are (a) robust against many environmental and genetic perturbations, but at the same time (b) sensitive to genetic changes in order to be 'reused' in different functional contexts [5]. Vertebrate limb development is a good example: during the development of an embryo, a well-regulated gene regulatory system controls the formation of the limbs; but the comparison of different vertebrate limbs shows that the same elements can be used, with slight variations, to

Copyright © 2002 John Wiley & Sons, Ltd.

build a wide variety of limbs, such as wings, legs and fins [11,14].

On the other hand, Featherstone and Broadie conclude from their degree distribution 'that the gene expression network consists of a single giant functional component rather than several subnetworks ... no subset of genes can be considered isolated from another' [4]. Rung *et al.* also address the question of how many components their graphs have. For a wide range of significance cut-offs they only find one major component, which consists of thousands of nodes, and no, or few, small other components.

Several explanations are possible for these differences, e.g. Maniatis and Reed emphasize the extensive degree of physical coupling among gene expression machines [8]. The main question is, how close are the simulated networks studied by Wagner to the networks studied by Rung *et al.* and Featherstone and Broadie? The graphs constructed by Rung *et al.* do have direct and indirect connections between some of the nodes. But there is not always a direct connection if there is an indirect connection between two genes. This is different in the examples given by Wagner and might account for the differences in the results. Furthermore, the power-law distribution used by Wagner for graph construction is considerably different from that found by Featherstone and Broadie and Rung *et al.* There may be even more differences: Maslov *et al.* compared protein-interaction networks and regulatory networks with randomized networks and found striking differences in the topology: the hubs of the 'real' networks are less likely to be connected to other hubs: '... links between highly connected proteins are systematically suppressed, whereas those between highly connected and low-connected pairs of proteins are favoured' [9]. They conclude that their results are '... consistent with compartmentalization and modularity characteristic of control of many cellular processes ... it suggests the picture of functional modules of the cell organized around individual hubs'. They propose that this reduces the cross-talk between functional modules of the cell and increases the robustness against perturbations.

Featherstone and Broadie are specifically addressing the question of buffering in gene regulatory networks. Why do so many gene deletions have no obvious phenotypic effect? They argue that a reason for this might be found in the topology of the network. Albert *et al.* have compared networks with a power-law degree distribution with networks having an exponential degree distribution and found that power-law networks are more robust towards random errors, but more vulnerable to targeted attacks [2]. They looked for the change in the diameter of the network if either random nodes were removed (error) or hubs were removed (attack). Thus, the structure of the network might explain the robustness against mutations of non-hubs, but would imply more serious effects when hubs are hit.

Featherstone and Broadie found stronger sequence conservation for hub genes than for non-hub genes. However, they only found a low correlation between the growth rate of the yeast mutants and the degree of the respective genes in the network, but obviously deletions of genes with the most severe effect, lethality, cannot be studied in these experiments.

Since the deletion of the hubs has only minor effects on the fitness of the yeast organism, Featherstone and Broadie suggest using yeast mutants with a reduced number of hub-genes as an ideal genetic background for deletion experiments, in order to reduce pleiotropic effects and to reduce interconnectivity. Yet when Rung *et al.* removed the hubs from their graphs, the major component did not break into several big components. The major component of the resulting graph was smaller, but the decrease in size was mainly due to single nodes falling off. There are only few more minor components with simple topologies. Only when removing 10% of all nodes, which leads to the loss of about 50% of all arcs, were several bigger components found at quite stringent thresholds for the expression data. It remains to be tested *in vivo* how many of the hub-genes can be removed without having a lethal effect on the yeast cell.

The study of gene deletion networks has led to interesting claims about the structure of gene regulatory networks. It remains to be seen how close networks built from gene deletion experiments are to the 'real' gene regulatory networks. Only one growth condition was tested by Hughes *et al.* and this limits the scope of the models built from that data. The integration of additional datasets, such as time course experiments, conditional mutants and chromatin immunoprecipitation experiments, might

Copyright © 2002 John Wiley & Sons, Ltd. 

help to improve the models [20]. Graph theory will be an important tool in helping us unravel the structure of the gene regulatory networks.

### Acknowledgements

We would like to thank Laurence Ettwiller and Helen Parkinson for critical reading of the manuscript and Johan Rung and Michael Lappe for fruitful discussions. Further, TS would like to thank the organizers and participants of the International Summer School 'From Genome to Life', Cargèse, for a stimulating and interesting time.

### References

1. Albert R, Barabási A-L. 2002. Statistical mechanics of complex networks. *Rev Mod Phys* **74**(47).
2. Albert R, Jeong H, Barabasi AL. 2000. Error and attack tolerance of complex networks. *Nature* **406**(6794): 378–382.
3. Cormen TH, Leiserson CE, Rivest RL. 1990. *Introduction to Algorithms*. MIT Press: Cambridge, MA.
4. Featherstone DE, Broadie K. 2002. Wrestling with pleiotropy: genomic and topological analysis of the yeast gene expression network. *Bioessays* **24**(3): 267–274.
5. Hartwell LH, Hopfield JJ, Leibler S, Murray AW. 1999. From molecular to modular cell biology. *Nature* **402**(6761 suppl): C47–C52.
6. Hughes TR, Marton MJ, Jones AR, *et al*. 2000. Functional discovery via a compendium of expression profiles. *Cell* **102**(1): 109–126.
7. Jeong H, Tombor B, Albert R, Oltvai ZN, Barabasi AL. 2000. The large-scale organization of metabolic networks. *Nature* **407**(6804): 651–654.
8. Maniatis T, Reed R. 2002. An extensive network of coupling among gene expression machines. *Nature* **416**(6880): 499–506.
9. Maslov S, Sneppen K. 2002. Specificity and stability in topology of protein networks. *Science* **296**(5569): 910–913.
10. McAdams HH, Shapiro L. 1995. Circuit simulation of genetic networks. *Science* **269**(5224): 650–656.
11. Ohuchi H, Takeuchi J, Yoshioka H, *et al*. 1998. Correlation of wing–leg identity in ectopic FGF-induced chimeric limbs with the differential expression of chick Tbx5 and Tbx4. *Development* **125**(1): 51–60.
12. Park J, Lappe M, Teichmann SA. 2001. Mapping protein family interactions: intramolecular and intermolecular protein family interaction repertoires in the PDB and yeast. *J Mol Biol* **307**(3): 929–938.
13. Rung J, Schlitt T, Brazma A, Freivalds K, Vilo J. 2002. Building and analysing genome-wide gene disruption networks. *Bioinformatics* **18**(suppl 2): S202–S210.
14. Ruvinsky I, Gibson-Brown JJ. 2000. Genetic and developmental bases of serial homology in vertebrate limb evolution. *Development* **127**(24): 5233–5244.
15. Schwikowski B, Uetz P, Fields S. 2000. A network of protein–protein interactions in yeast. *Nature Biotechnol* **18**(12): 1257–1261.
16. Smolen P, Baxter DA, Byrne JH. 2000. Modeling transcriptional control in gene networks-methods, recent results, and future directions. *Bull Math Biol* **62**(2): 247–292.
17. Thieffry D, Huerta AM, Perez-Rueda E, Collado-Vides J. 1998. From specific gene regulation to genomic networks: a global analysis of transcriptional regulation in *Escherichia coli*. *Bioessays* **20**(5): 433–440.
18. Thieffry D, Romero D. 1999. The modularity of biological regulatory networks. *Biosystems* **50**(1): 49–59.
19. Wagner A. 2002. Estimating coarse gene network structure from large-scale gene perturbation data. *Genome Res* **12**(2): 309–315.
20. Wyrick JJ, Young RA. 2002. Deciphering gene expression regulatory networks. *Curr Opin Genet Dev* **12**(2): 130–136.

Copyright © 2002 John Wiley & Sons, Ltd. 
